# Investigation on Mechanical Properties, Damage Forms, and Failure Mechanisms of Additively Manufactured Schoen Gyroid TPMS Porous Structures Under Compressive Load

**DOI:** 10.3390/ma19010149

**Published:** 2025-12-31

**Authors:** Yang Hou, Xuanming Cai, Wei Zhang, Bin Liu, Zhongcheng Mu, Junyuan Wang, Linzhuang Han, Wenbo Xie, Heyang Sun

**Affiliations:** 1 School of Materials Science and Engineering, North University of China, Taiyuan 030051, China; houyang1010@163.com (Y.H.); liubin3y@nuc.edu.cn (B.L.); 2 School of Aerospace Engineering, North University of China, Taiyuan 030051, China; h13082018371@163.com; 3 School of Astronautics, Harbin Institute of Technology, Harbin 150080, China; zhdawei@hit.edu.cn; 4 School of Aeronautics and Astronautics, Shanghai Jiao Tong University, Shanghai 200240, China; muzhongcheng@sjtu.edu.cn; 5 School of Mechanical Engineering, North University of China, Taiyuan 030051, China; wangjy@nuc.edu.cn; 6 Power China Kunming Engineering Corporation Limited, Kunming 650051, China; xiewbhit@foxmail.com; 7 Shijiazhuang Campus, Army Engineering University of PLA, Shijiazhuang 050003, China; flyn4983@163.com

**Keywords:** additive manufacture, damage mode, mechanical testing, compressive deformation, numerical simulation

## Abstract

To address the conflicting demands of lightweight materials and high load-bearing capacity in high-end fields such as aerospace and biomedical engineering, there is an urgent need to conduct research on the mechanical behavior and response mechanism of porous titanium alloy structures. In this paper, a combination of experimental testing, numerical simulation, and theoretical analysis was employed to conduct the research. A titanium alloy porous structure with different porosities was constructed based on classical three-period minimal surface optimization, and its preparation was completed using advanced selective laser melting technology. A multidimensional characterization experimental device was established to accurately obtain its mechanical performance data. It was found that the mechanical behavior of the structures is insensitive to loading rates, but more sensitive to their structural volume fraction. The quantitative characterization of microstructure damage and fracture morphology, as well as the identification of failure modes, indicates that the microstructure damage of the porous metal exhibits a ductile–brittle synergistic damage characteristic. By combining high-precision numerical simulation technology, the damage modes and damage evolution laws of porous metal structures in titanium alloys were comprehensively elucidated. By analyzing energy dissipation and constructing evaluation indicators for energy absorption efficiency, the energy absorption characteristics of the porous metal structure were elucidated, and the interaction behavior and correlation mode between the platform stress and the structural volume fraction of the porous metal structure were accurately described.

## 1. Introduction

The porous structure of a titanium alloy, with its unique lightweight characteristics and excellent energy absorption capacity, has shown great potential for applications in high-end fields such as aerospace, biomedical implant devices, and national defense protection [1,2,3,4,5]. Under low strain rate loading conditions, in-depth exploration of the mechanical behavior and damage mechanism of the structural system not only helps to reveal the synergistic bearing law between the pore structure and the matrix material at the microscale [6,7,8], but also accurately constructs a multi-scale constitutive model that includes elastic-plastic deformation, local buckling, and progressive damage processes [9,10,11]. It can also provide key theoretical support for the optimization design, life prediction, and failure prevention of structural components in extreme service environments [12,13,14]. With the rapid increase in demand for high-performance lightweight materials in modern engineering technology, analyzing the mechanical response mechanism of titanium alloy porous structures at low strain rates has become a core issue in breaking through material performance bottlenecks and promoting technological innovation in related fields [15,16,17]. It has significant strategic significance and practical urgency for enhancing the independent innovation capability and international competitiveness of the high-end equipment manufacturing industry.

In current research, certain achievements have been accumulated regarding the mechanical behavior and damage mechanism of porous structures of titanium alloys at low strain rates [18,19,20,21], but there are still many areas that need to be improved. On the one hand, in the study of mechanical behavior, although the influence of factors such as porosity, pore size distribution, and pore structure morphology on their mechanical properties has been revealed [22,23,24,25,26,27], the research on the damage mode and evolution expansion mode of complex porous structures, especially curved porous metal structures, under low strain rate conditions is still relatively weak [28,29,30,31,32]. For example, in the aerospace field, aircraft structures bear the responsibility of protection, and existing research is difficult to fully describe the mechanical response and protection mechanism of titanium alloy porous structures, lacking a systematic and in-depth understanding [33,34,35]. On the other hand, in the exploration of damage mechanisms, although the main forms of damage, such as pore collapse, matrix cracking, and interface debonding, have been identified, the influence of material microstructure evolution on damage development at the microscale has not been fully analyzed [36,37,38]. Existing research mostly focuses on the performance verification of single TPMS structures, lacking composite optimization design methods for engineering applications. This study combines topology optimization and structural gradient design concepts to establish a dual-dimensional optimization strategy of “density gradient + structural gradient”, which solves the engineering bottleneck of traditional TPMS structures that are difficult to balance lightweight and high load-bearing requirements. In addition, in terms of research methods, although experiments and numerical simulations complement each other [39,40,41], there is still room for improvement in their collaborative innovation. For example, the precise integration of characterization methods and numerical simulation models in experiments, as well as how to construct high-precision numerical simulation research, all require further deepening of multidimensional research methods to solve them. This will comprehensively and accurately reveal the mechanical behavior and damage mechanism of titanium alloy porous structures at low strain rates, providing a solid theoretical basis and technical support for their engineering applications in various key fields.

In this paper, a systematic study is conducted on the mechanical behavior and response mechanism of porous metal structures in titanium alloys. Based on the principles of three-period minimal surfaces and fractal geometry, a multi-component partition configuration is constructed to achieve parameterized control of porosity and connectivity. Obtain mechanical response data under different loading rates and stress states through quasi-static compression tests. Establish high-precision numerical simulation research techniques to deeply analyze the forms of load action and deformation localization phenomena. By analyzing energy dissipation and constructing evaluation indicators for energy absorption efficiency, this study explores the influence of pore structure parameters on energy absorption performance, revealing its energy absorption mechanism and failure mode.

## 2. Experimental Materials and Specimens

### 2.1. Experimental Materials

**Figure 1:**
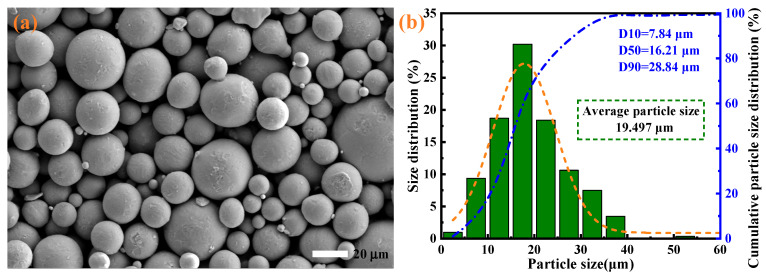
(**a**) Microstructure of Ti6Al4V powder particles in this paper and (**b**) distribution of particle sizes.

The Ti6Al4V powder used in this paper belongs to the (α + β)-type titanium alloy, with the main metal elements being Ti, Al, V, and a small amount of other elements. Its detailed composition is shown in Table 1. Figure 1a shows the microstructure of Ti6Al4V powder particles, which are spherical in shape, smooth on the surface, and have good flowability. The particle size distribution of Ti6Al4V powder is shown in Figure 1b, with an average particle size of 19.497 μm and a density of approximately 4.47 g/cm^3^, demonstrating excellent comprehensive mechanical properties.

### 2.2. Experimental Specimen Design

In three-dimensional space, three-dimensional periodic minimum surfaces (TPMS) with periodic symmetry can be used to simulate and design materials with special properties, such as high-strength and lightweight materials. These surfaces can help understand the relationship between the microstructure and macroscopic properties of materials. The surface of a TPMS is smooth, and the pores are highly connected, and the overall structure is precisely controlled by implicit functions, making it an excellent solution for designing and constructing porous structures [42,43]. The Schoen Gyroid surface based on TPMS has significant advantages in constructing porous metal structures, expressed in the following form:

(1) $$\begin{array}{l} G(x,y,z)=\cos\left(\frac{2\pi }{a}\cdot x\right)\sin\left(\frac{2\pi }{a}\cdot y\right)+\cos\left(\frac{2\pi }{a}\cdot y\right)\sin\left(\frac{2\pi }{a}\cdot z\right)+\\ \;\;\;\;\;\;\;\cos\left(\frac{2\pi }{a}\cdot z\right)\sin\left(\frac{2\pi }{a}\cdot x\right)-t=0 \end{array}$$

**Figure 2:**
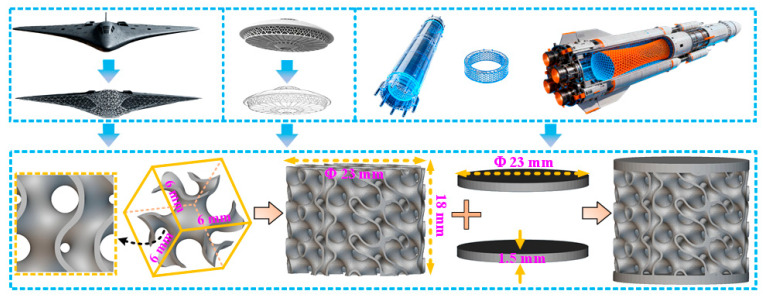
Main formation process of the porous structure for Schoen Gyroid TPMS.

In the equation, *a* is the length of the unit cell, and the parameter *t* controls the surface volume of the Schoen Gyroid function. Figure 2 shows the main process of optimizing the design of Ti6Al4V porous structure specimens in this study. Firstly, a cubic basic cell structure was determined in three-dimensional space, with dimensions of 6 mm × 6 mm × 6 mm. Then, use this cubic unit cell as a repeating unit for a 3 × 3 × 3 array stacking along the X, Y, and Z axes to construct a porous core structure with a theoretical size of 18 mm × 18 mm × 18 mm. To meet the requirements of compressive testing, the porous core was further processed into a cylindrical shape with a diameter of 23 mm, and the height was retained at 18 mm. Finally, two circular end caps (top and bottom) with a diameter of 23 mm and a uniform thickness of 1.5 mm were used to encapsulate the cylindrical porous core, ensuring overall structural balance and completing the construction of the specimen with a total height of 21 mm. Considering the lightweight and impact resistance properties of the Ti6Al4V porous structure, three reasonable structural volume fractions were optimized by exclusively adjusting the wall thickness of the TPMS framework with no other variables introduced, namely 9.43%, 15.83%, and 22.15%, and defined as the A1 structure, A2 structure, and A3 structure, respectively. After the completion of the specimen structure design, selective laser melting technology was used for preparation.

The laser processing and post-heat treatment parameters for fabricating the specimens are specified as follows: The laser power and scanning speed for the core printing area were set to 180 W and 1000 mm/s, respectively. The scanning strategy adopted line filling with a hatch spacing of 0.180 mm, a rotation angle of 67° between adjacent layers, and the SFP (Selective Fractional Partitioning) zonal scanning sequence. The building direction was aligned such that the cylindrical axis of the specimen was parallel to the *Z*-axis of the equipment. Trapezoidal support structures were employed, with parameters including 4 offset layers, an offset distance of 0.080 mm, and a minimum bandwidth of 6.000 mm. For the skin control parameters, the top skin was filled with a spacing of 0.160 mm for 1 pass with an overlap of 0.300 mm; the bottom skin was filled with a spacing of 0.120 mm for 1 pass with an overlap of 0.300 mm; and the detail transition distance was 2.000 mm. The specific heat treatment parameters were as follows: holding at 320 °C for 2 h with a heating rate of 5 °C/min, followed by furnace cooling to room temperature.

## 3. Experimental and Numerical Simulation Research Methods

### 3.1. Experimental Methods

This study conducted quasi-static loading experiments on Ti6Al4V porous metal structures using the SHIMADZU universal testing machine (WDW-100, Shimadzu (China) Co., Ltd., Jinan, China). The testing machine has a maximum loading capacity of 200 kN and can stably achieve multi-scale time displacement loading control. The loading rate range of the test specimens in the study is 1~100 mm/min. The force time curve data during the loading process is monitored and collected in real time through a force sensor integrated into the testing machine. To achieve accurate measurement of the deformation state during the loading process of the specimen, a non-contact full-field deformation optical measurement technology—Digital Image Correlation (DIC)—is used for monitoring. In the specific operation, irregular speckle patterns are uniformly sprayed at the end of the loading head (near the specimen area) of the testing machine as a deformation information carrier. The displacement field of the entire field is analyzed through image processing algorithms, and the overall strain value during the loading process is accurately calculated based on the initial length of the specimen. To analyze the failure mode of Ti6Al4V porous metal structure during loading, a multi-directional synchronous monitoring system was built using two Manta G-917B cameras (Allied Vision Technologies GmbH, Stadtroda, Thuringia, Germany) to record the deformation and failure process of the specimen in real-time from two orthogonal perspectives. This experimental scheme achieves multi-physics field quantification analysis of quasi-static loading behavior of porous metal structures through the synergistic coupling of the mechanical loading system and the optical measurement system.

### 3.2. Numerical Simulation Method

#### 3.2.1. Finite Element Model

In this study, HyperMesh software (Version: 2021, Altair Engineering, Inc., Troy, MI, USA) was employed for meshing the Ti6Al4V porous metallic structures. A systematic mesh convergence analysis was conducted using the specimen with a volume fraction of 15.83% as the benchmark: five mesh sizes ranging from 0.05 mm to 0.25 mm were compared, with the axial compressive peak stress serving as the convergence criterion (allowable error ≤ 2%). The results indicated that the relative error between the 0.1 mm mesh and the 0.05 mm high-precision reference mesh was merely 0.34%. Considering both accuracy and efficiency, the 0.1 mm mesh was determined as the final mesh size. After optimization via the Laplacian smoothing algorithm, the model was imported into the ABAQUS software (Version: 2021, Dassault Systèmes Simulia Corp., Providence, RI, USA) and discretized using 0.1 mm tetrahedral elements. The augmented Lagrangian method was adopted for contact modeling between the end caps and the porous region. Based on experimental conditions, a systematic investigation was carried out on the axial compressive mechanical responses of Ti6Al4V porous structures with three different volume fractions.

#### 3.2.2. Material Constitutive Model and Related Parameters

The Johnson–Cook constitutive model can accurately describe the behavioral characteristics of metal materials in complex mechanical environments. This model constructs a comprehensive and accurate material constitutive relationship by considering factors such as strain rate strengthening, temperature softening, and plastic deformation of the material, which can accurately describe the mechanical response of the material under large deformation conditions [44,45,46,47]. Its expression is as follows:

(2) $$\sigma =(A+B\varepsilon_{p}^{n})(1+C\ln{\dot{\varepsilon }}_{p}^{*})(1-T^{*m})$$

In this formula system, *A* represents the yield strength parameter, and its numerical value directly affects the stress level at which the material begins to undergo plastic deformation. *B*, as a model parameter, plays a key role in constructing the material mechanical behavior model. *n* is the hardening index, which is used to characterize the degree to which the strength of a material increases with the amount of deformation during plastic deformation. *C* is a strain rate sensitive parameter, which reflects the sensitivity of material flow stress to changes in strain rate. *m* is the temperature softening parameter, which reflects the degree of influence of temperature increase on the weakening of material strength.

Among them, *σ* is the equivalent flow stress, which is an important indicator to measure the material’s ability to resist plastic deformation under complex stress states, and *ε_p_* is the equivalent plastic strain, used to quantify the amount of plastic deformation that has occurred in the material. ${\dot{\varepsilon }}_{p}^{*}$ is the dimensionless equivalent plastic strain rate, ${\dot{\varepsilon }}_{p}^{*}={\dot{\varepsilon }}_{p}/{\dot{\varepsilon }}_{0}$, ${\dot{\varepsilon }}_{p}$ is the actual equivalent plastic strain rate, and ${\dot{\varepsilon }}_{0}$ is the reference strain rate. T* is the dimensionless temperature, $T^{*}=(T-T_{r})/(T_{m}-T_{r})$, where *T*, *T_r_*, and *T_m_* are the current temperature, reference temperature, and melting point temperature, respectively. The specific values of all the above parameters are shown in Table 2.

#### 3.2.3. Material Failure Criteria and Related Parameters

The Johnson–Cook fracture criterion is an empirical gold standard constitutive model applicable to characterize the fracture behavior of materials under large plastic deformation conditions. This criterion integrates the synergistic mechanism of multidimensional mechanical parameters, such as stress triaxiality, cumulative plastic strain, and strain rate effect on the material failure process by constructing a fracture criterion system that couples multiple physical fields. The core lies in establishing a fracture prediction equation that covers stress state characterization parameters, plastic deformation damage threshold, and strain rate sensitivity coefficient through dimensionless processing and phenomenological modeling methods, thereby achieving a quantitative description of material cracking and instability propagation behavior under complex loading conditions [48,49,50,51]. This model has wide engineering application value in the field of dynamic failure analysis of metal materials, while it is also applicable to quasi-static scenarios for specific structured materials; for the Schoen Gyroid topological Ti-6Al-4V lattice in this study, buckling and collapse of struts during compression will induce local instantaneous strain rate elevation. Conventional elastoplastic models fail to capture this response, whereas the strain rate-dependent term of the Johnson–Cook model can accurately characterize this feature. The model parameters are adjusted to adapt to quasi-static conditions: this modification not only retains the model’s advantage in describing complex stress states, but also realistically reproduces the entire process of “strut deformation-damage accumulation-fracture” of the lattice structure, resulting in better physical consistency than simple elastoplastic models. Its expression form is as follows:

(3) $$\varepsilon_{f}=[D_{1}+D_{2}\exp(D_{3}\sigma^{*})](1+D_{4}\ln{\dot{\varepsilon }}_{p}^{*})(1+D_{5}T^{*})$$

In the equation, *ε_f_* is the fracture strain, σ* is the stress triaxiality (σ* = *σ_m_*/*σ_Mises_*, *σ_m_* is the hydrostatic pressure, and *σ_Mises_* is the equivalent stress), and *D*_1_~*D*_5_ are the material model parameters, with specific values shown in Table 3.

## 4. Results and Discussion

### 4.1. Experimental Results

**Figure 3:**
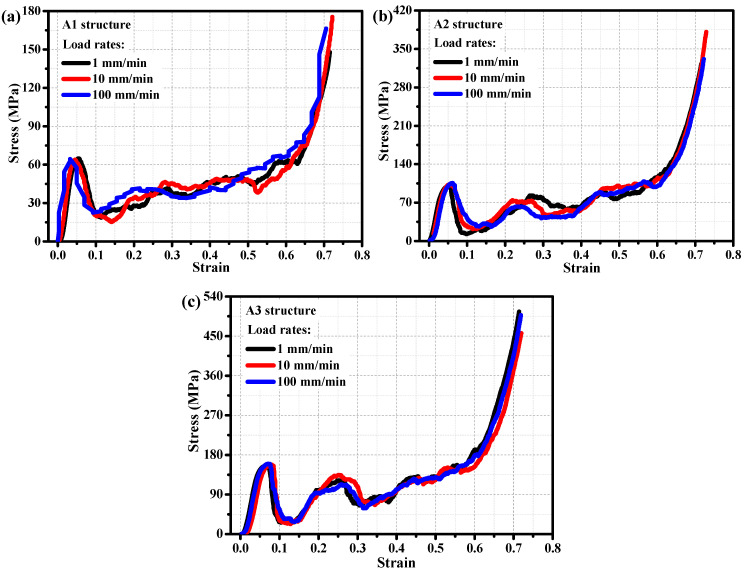
At different loading rates, stress–strain curves of porous metal structures for (**a**) A1 structure, (**b**) A2 structure, and (**c**) A3 structure.

To investigate the effect of different loading rates on the mechanical behavior of porous structures in titanium alloys, and to further study their mechanical response characteristics at loading rates of 1 mm/min, 10 mm/min, and 100 mm/min. Figure 3 shows the stress–strain relationship of three different volume fraction titanium alloy porous structures under different loading rates. From Figure 3a, it can be seen that the stress–strain curves of the A1 structure are relatively similar at different loading rates, and its bearing strength (about 64.5 ± 1.9 MPa) is also relatively similar, indicating that the mechanical behavior of the A1 structure at different loading rates is not sensitive to loading rates. Figure 3b shows that the mechanical behavior of the A2 structure at different loading rates also exhibits insensitivity to loading rates, with a bearing strength of approximately 102.9 ± 3.1 MPa, significantly higher than that of the A1 structure with a smaller volume fraction. The A3 structure also exhibits similar mechanical properties (Figure 3c), and its mechanical properties at different loading rates are not sensitive to loading rates. However, the bearing strength of the A3 structure with the highest volume fraction (157.9 ± 4.7 MPa) is significantly higher than that of the A2 structure with a lower volume fraction. To verify the consistency of the mechanical properties of a single structure, the Shapiro–Wilk test was used to confirm that the data followed a normal distribution (*p* > 0.05), and the Grubbs test was employed to eliminate outliers. The coefficient of variation (CV) for key mechanical parameters (such as elastic modulus and yield strength) of structures A1, A2, and A3 was calculated to be ≤3.4%, which is significantly lower than the acceptable threshold of CV < 5% in similar material mechanical research, indicating good internal consistency of the data. From the analysis of stress–strain curves of porous titanium alloy structures under different loading rates, it can be concluded that the mechanical behavior of the porous titanium alloy structure is insensitive to loading rates, but more sensitive to its structural volume fraction.

**Figure 4:**
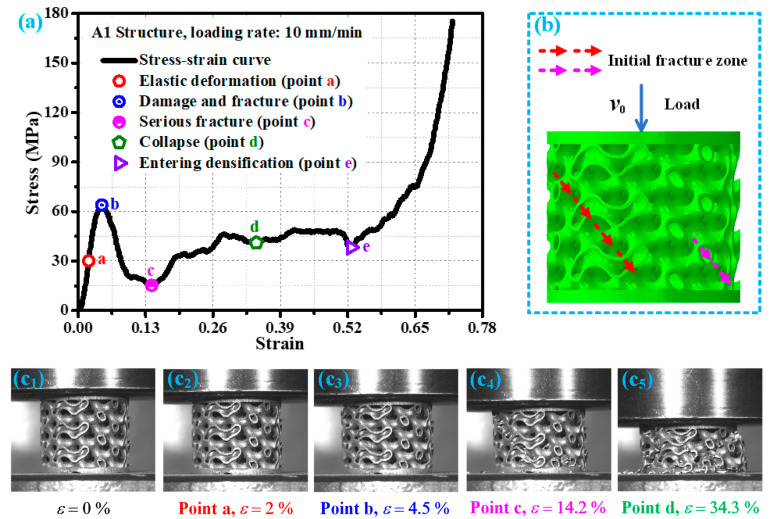
Mechanical behavior of A1 structure under loading rate of 10 mm/min: (**a**) key response points of the stress—strain curve; (**b**) schematic diagram of damage and fracture direction; (**c_1_**–**c_5_**) damage forms of corresponding structural key response points in experimental research.

Figure 4 shows the stress–strain curve of the A1 structure at a loading rate of 10 mm/min and the damage modes corresponding to its key points. According to the analysis in Figure 4a, it can be seen that in the early stage of loading, the required external load of the A1 structure gradually increases with the increase in strain, and its stress–strain relationship shows a linear relationship (as shown in point a in Figure 4a), which is manifested as elastic deformation. The deformation generated by the A1 structure in this elastic stage is elastic deformation, and the deformation mode corresponding to point a in the stress–strain curve is shown in Figure 4(c_2_). As the loading continues, while the load exceeds the bearing strength of the A1 structure (point b in Figure 4a), the structure begins to produce damage cracks (Figure 4(c_3_)), and the initial damage fracture location is indicated by the red arrow in Figure 4b. Continuing to load, the damage cracks of the A1 structure rapidly propagate along the stress weak position, and its resistance to failure gradually decreases. At this time, its stress–strain curve also sharply decreases. With the continuous action of compressive load, a new initial damage fracture zone appeared in the A1 structure damage crack during the propagation process (as shown by the pink arrow in Figure 4b). At this time, the stress–strain curve of A1 structure showed a low point (point c in Figure 4a), and its damage was already quite severe, as shown in Figure 4(c_4_). Continuing to load, the initial damage fracture area is temporarily compacted. Although the A1 structure has a certain degree of resistance to damage during this process, its damage intensifies with the loading action. As the strain is about 34.3% (point d in Figure 4a), the A1 structure is crushed, and multiple cumulative damages have occurred, as shown in Figure 4(c_5_). As the external load continues to act, the A1 structure gradually becomes compacted and approaches the densification stage. When its strain is about 52.7%, the A1 structure reaches the densification stage (point e in Figure 4a). The mechanical response and response mode of the A1 structure under loading rates of 1 mm/min and 100 mm/min are basically similar to those under loading rates of 10 mm/min.

**Figure 5:**
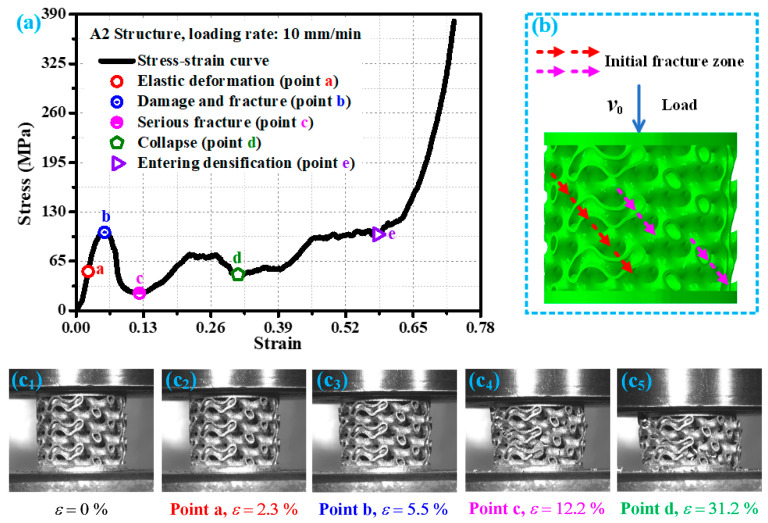
Mechanical behavior of A2 structure under loading rate of 10 mm/min: (**a**) key response points of the stress—strain curve; (**b**) schematic diagram of damage and fracture direction; (**c_1_**–**c_5_**) damage forms of corresponding structural key response points in experimental research.

Figure 5 shows the stress–strain curve of the A2 structure at a loading rate of 10 mm/min and the corresponding damage modes of the A2 structure at key points. According to the analysis in Figure 5, during the initial loading stage, the A2 structure undergoes elastic deformation. The point a (strain of approximately 2.3%) on the stress–strain curve in Figure 5a is in the elastic stage, and its deformation form is shown in Figure 5(c_2_). As the loading continues, the internal stress of the A2 structure gradually increases. When the external load exceeds the bearing strength of the A2 structure (point b in Figure 5a), damage fracture begins to occur (Figure 5(c_3_)), and the damage fracture area is shown by the red arrow in Figure 5b. Continuing to load, the damage and fracture of the A2 structure continue to expand, and new initial damage and fracture areas are generated during the expansion process (pink arrow in Figure 5b). As the load continues to act, the A2 structure experiences temporary compaction and severe damage, as shown in Figure 5(c_4_). At this point, the stress–strain curve shows a low point (point c in Figure 5a). Continuing to load, the damage and fracture of the A2 structure are becoming increasingly severe. While the strain is about 31.2% (point d in Figure 5a), the A2 structure is crushed, and the accumulated damage is more severe, as shown in Figure 5(c_5_). As the compressive load continues to act, the A2 structure is gradually compacted. When the strain is about 58.1%, the A2 structure begins to enter the densification stage (point e in Figure 5a). The mechanical response and response mode of the A2 structure at loading rates of 1 mm/min and 100 mm/min are basically similar to those at loading rates of 10 mm/min.

**Figure 6:**
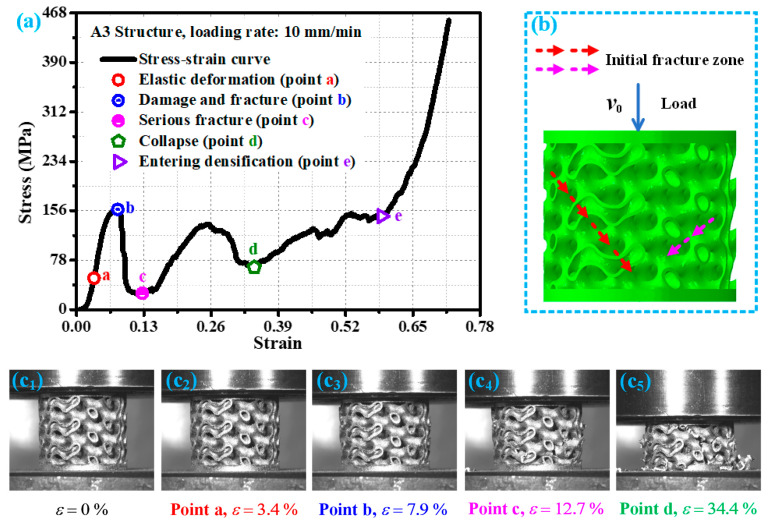
Mechanical behavior of A3 structure under loading rate of 10 mm/min: (**a**) key response points of the stress—strain curve; (**b**) schematic diagram of damage and fracture direction; (**c_1_**–**c_5_**) damage forms of corresponding structural key response points in experimental research.

Figure 6 shows the stress–strain curve of the A3 structure at a loading rate of 10 mm/min and the corresponding damage modes of the A3 structure at key points. At the beginning of loading, the A3 structure exhibits elastic stage mechanical behavior characteristics, with a linear relationship between stress–strain (point a in Figure 6a), and its elastic deformation is shown in Figure 6(c_2_). When the external load exceeds the bearing limit of the A3 structure (point b in Figure 6a), it begins to undergo damage and fracture (Figure 6(c_3_)), as indicated by the red arrow in Figure 6b. As the load continues to act, the damage and fracture of the A3 structure continue to expand, and new damage fractures are generated during this expansion process, with their positions and directions indicated by the pink arrows in Figure 6b. With the temporary completion of damage fracture propagation and continuous loading, the A3 structure exhibits temporary compaction, and its stress–strain curve shows a low point (point c in Figure 6a). At this point, the A3 structure has suffered severe damage and fracture (as shown in Figure 6(c_4_)), and temporary compaction has also occurred. As the load continues to act, the damage to the A3 structure intensifies. While the strain is about 34.4% (point d in Figure 6a), the A3 structure is crushed, and severe cumulative damage has already occurred, as shown in Figure 6(c_5_). Continuing to load, the A3 structure gradually approaches the densification stage. When the strain is about 58.9%, the A3 structure begins to enter the densification stage. The mechanical response and response mode of the A3 structure under loading rates of 1 mm/min and 100 mm/min are basically similar to those under loading rates of 10 mm/min.

### 4.2. Macroscopic Structural Damage Failure Mechanism

**Figure 7:**
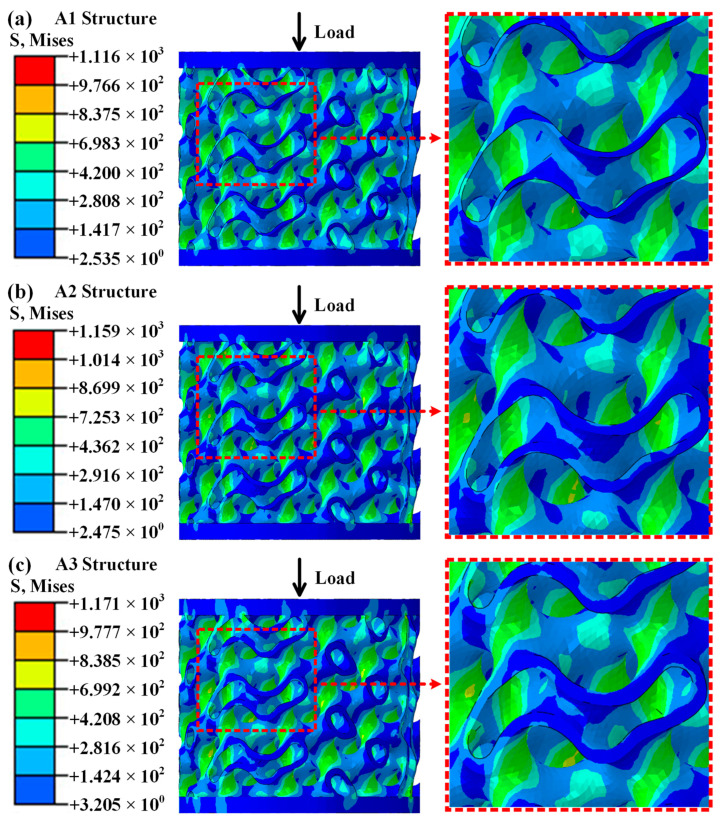
Load-bearing state of the porous metal structures during the elastic stage at a loading rate of 10 mm/min: (**a**) stress state exhibited by the A1 structure at strain *ε* = 2%; (**b**) stress state exhibited by the A2 structure at strain *ε* = 2.3%; (**c**) stress state exhibited by the A3 structure at strain *ε* = 3.4%.

In order to further explore the damage mode of porous structures in titanium alloys and achieve real-time multi-angle monitoring of their damage forms, high-precision numerical simulation research was carried out to reveal the damage mechanism of porous structures in titanium alloys. Figure 7 shows the load-bearing state of the porous structure of titanium alloy during the elastic stage at a loading rate of 10 mm/min. Figure 7a shows the stress state exhibited by the A1 structure at strain *ε* = 2%. Based on the numerical simulation results, it can be concluded that the overall stress distribution is relatively uniform under axial compression load, with no obvious stress concentration phenomenon. Figure 7b,c show the stress states exhibited by the A2 structure at a strain of 2.3% and the A3 structure at a strain of 3.4%, respectively. The overall stress distribution is relatively uniform, with no obvious stress concentration phenomenon. Compared with the experimental research results (Figure 4(c_2_), Figure 5(c_2_), and Figure 6(c_2_)), there is no significant difference in the structural elastic deformation form in the numerical simulation results.

**Figure 8:**
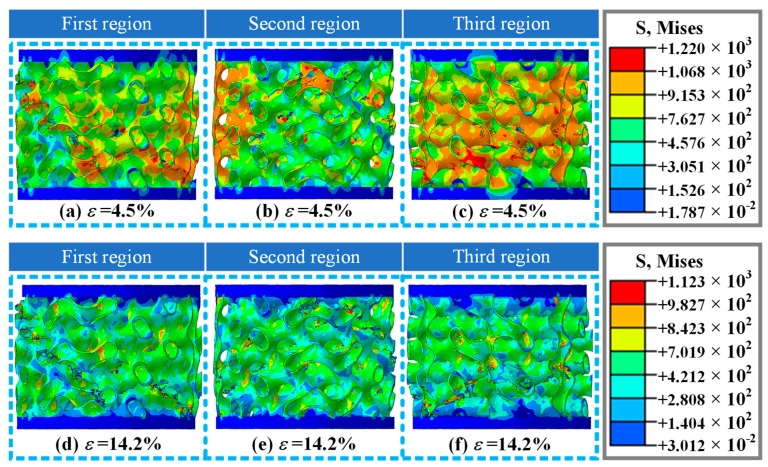
Damage and fracture forms of A1 structure at three different orientations in numerical simulation results at a loading rate of 10 mm/min: (**a**–**c**) deformation and failure modes in three different directions at strain *ε* = 4.5%, respectively; (**d**–**f**) deformation and failure modes in three different directions at strain *ε* = 14.2%, respectively.

Figure 8 shows the damage and fracture forms of the A1 structure at different orientations in numerical simulation when the strain is 4.5% and 14.2%. Figure 8a shows the damage and failure mode of the A1 structure at a strain of 4.5%, which is similar to the experimental results (Figure 4(c_3_)). At the same time, structural damage forms in other directions (Figure 8b,c) were provided, with multiple locations experiencing damage and fracture. This damage pattern also provides an important reference for revealing the mechanism of structural damage. Figure 8d–f show the deformation and failure modes in different directions of the A1 structure as the strain is 14.2% in the numerical simulation results. The numerical simulation study with the same orientation as the experimental research shows a damage mode (Figure 8d) that is basically consistent with the experimental research results (Figure 4(c_4_)). Multiple damage fractures occurred in other directions under the same strain, providing important damage library data for the study of its structural damage mechanism.

**Figure 9:**
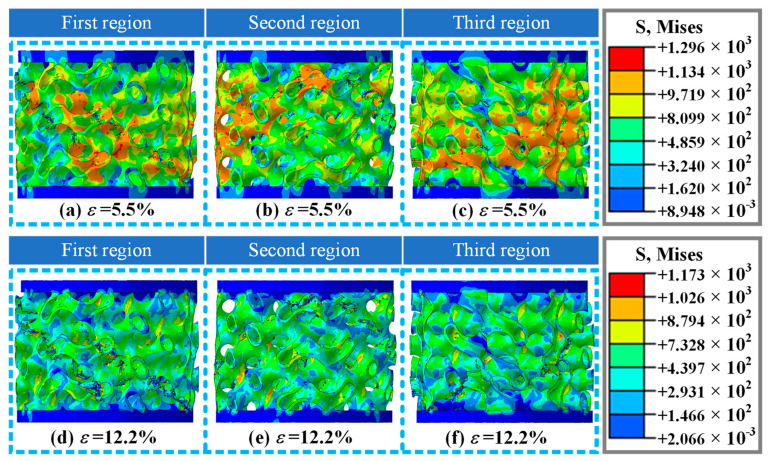
Damage and fracture forms of A2 structure at three different orientations in numerical simulation results at a loading rate of 10 mm/min: (**a**–**c**) deformation and failure modes in three different directions at strain *ε* = 5.5%, respectively; (**d**–**f**) deformation and failure modes in three different directions at strain *ε* = 12.2%, respectively.

Figure 9 shows the damage and fracture forms of the A2 structure at different orientations in numerical simulation, while the strain is 5.5% and 12.2%. As the strain is 5.5%, compared with the experimental results, the damage and fracture mode of the A2 structure in the numerical simulation (Figure 9a) is similar to the experimental results (Figure 5(c_3_)), including the fracture area, damage propagation mode, etc. The numerical simulation results also provide other different directional damage modes of the A2 structure (Figure 9b,c), including fracture zone, fracture mode, stress distribution form, etc. As the strain is 12.2%, the numerical simulation results of the A2 structure damage fracture form shown in Figure 9d show two obvious fracture zones, which are basically consistent with the experimental phenomenon (Figure 5(c_4_)). Numerical simulation studies have also explored the damage and fracture forms of A2 structure in different orientations under this strain (Figure 9e,f), including fracture zones, stress distribution patterns, etc., providing important reference for a comprehensive understanding of the damage and failure forms of A2 structure.

**Figure 10:**
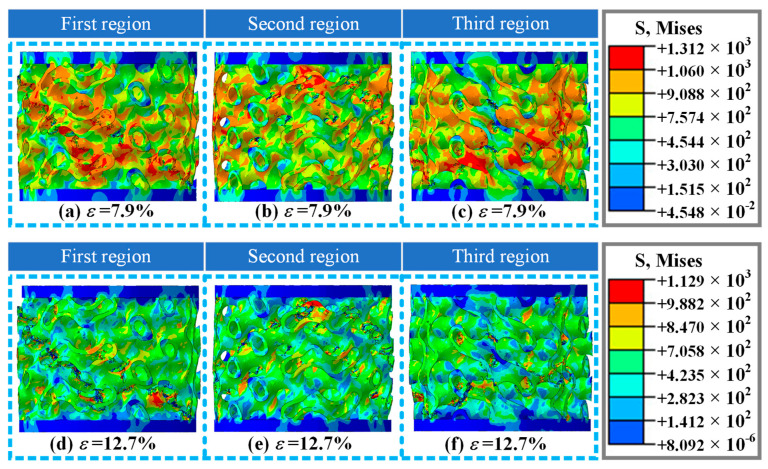
Damage and fracture forms of A3 structure at three different orientations in numerical simulation results at a loading rate of 10 mm/min: (**a**–**c**) deformation and failure modes in three different directions at strain *ε* = 7.9%, respectively; (**d**–**f**) deformation and failure modes in three different directions at strain *ε* = 12.7%, respectively.

Figure 10 shows the damage and fracture forms of the A3 structure in different orientations under strains of 7.9% and 12.7% in numerical simulation. While the strain was 7.9%, a damage fracture occurred in the A3 structure, and the fracture form and position were consistent with the experimental results (Figure 6(c_3_)). The numerical simulation study also investigated the damage morphology of the A3 structure at different orientations under this strain (Figure 10b,c), including the fracture zone and high-risk stress distribution. As the strain is 12.7%, the damage intensifies, and two obvious shear fracture zones appear in the A3 structure (Figure 10d), which are basically consistent with the experimental results of the failure mode (Figure 6(c_4_)). The numerical simulation study also investigated the damage morphology of the A3 structure at different orientations under this strain (Figure 10e,f), including the fracture zone, fracture orientation, stress distribution form, etc. Based on the numerical simulation studies of A1, A2, and A3 structures, it is shown that this study is accurate and effective. It can be used to accurately describe the mechanical behavior of A1, A2, and A3 structures under axial loading conditions, and reveal the damage and fracture mechanisms of three thin-walled structures with different volume fractions.

### 4.3. Microscopic Structural Damage Mode

**Figure 11:**
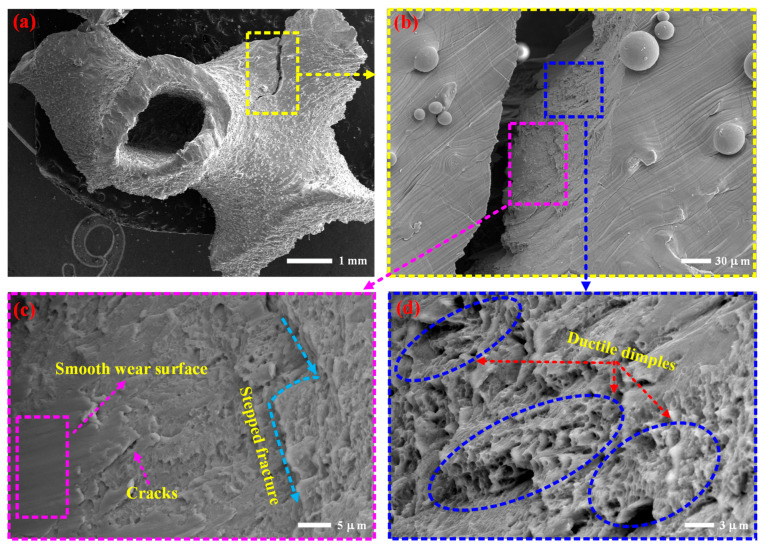
Microstructure damage morphology of the shear fracture zone: (**a**,**b**) it was found that there were fracture cracks on the fracture surface; (**c**) a stepped fracture mode with a smooth wear surface; (**d**) small and deep ductile dimples were also found in the shear fracture surface.

Figure 11 shows the microstructure damage morphology of the shear fracture zone in the specimen. Shear action is the main cause of macroscopic structural damage and fracture in specimens, and analyzing its microscopic structural damage mode can provide an important reference for the study of its damage mechanism. Analyzing the microstructure morphology of the shear fracture zone in Figure 11a,b, it was found that there were fracture cracks on the fracture surface, a stepped fracture mode, and a smooth wear surface, as shown in Figure 11c. These mechanical behaviors are closely related to its shear action. Small and deep ductile dimples were also found in the shear fracture surface (Figure 11d), with a width of approximately 1 μm. Some even experienced stacking and overlapping, indicating that the material underwent plastic deformation before fracture.

**Figure 12:**
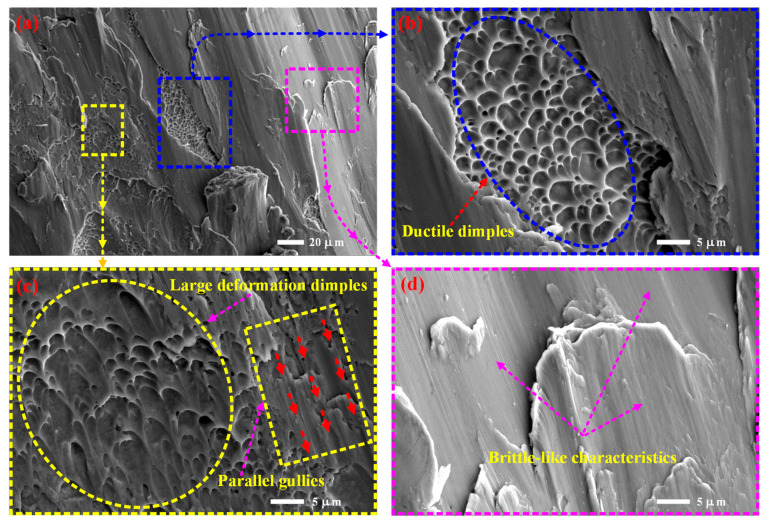
There is diversity in the forms of damage on the shear plane of the specimen: (**a**,**b**) a group of tough dimples with relatively large overall dimensions and varying sizes; (**c**) large deformation dimples and parallel gullies; (**d**) brittle-like characteristic mechanical behavior.

When conducting microscopic structural damage morphology analysis, as shown in Figure 12, it was found that there is diversity in the forms of damage on the shear plane of the specimen. Firstly, a group of tough dimples was also discovered, with varying sizes of dimples (mainly distributed in the range of 0.7 μm to 5 μm), but the overall size is relatively large, as shown in Figure 12a,b. In the nearby position, there is also a group of ductile dimples, which are relatively shallow but have a larger size along the tangential direction of the section and are greatly stretched. The dimples are parabolic in shape (Figure 12c), indicating that the position has undergone significant plastic deformation. At the same time, parallel grooves consistent with the direction of its large plastic deformation were found in the adjacent area, which is closely related to the coupling effect of compression/shear. In addition to the toughness mechanical properties, a large area of brittle-like characteristic fracture mechanics behavior was found on the fracture surface, as shown in Figure 12d, indicating that damage fracture occurred in this area at extremely low strain, resulting in brittle fracture. By combining existing morphological features with macroscopic mechanical parameters such as yield strength and compressive strength, a qualitative rule is proposed that “the increase in toughness density corresponds to an enhancement in plastic deformation ability, while the expansion of brittle-like characteristics regions leads to a decrease in fracture energy”. The comprehensive analysis of microstructure damage and fracture morphology shows that the damage form of the porous metal microstructure has a dual damage characteristic of toughness/brittleness. Of course, there are objective limitations in the observation of structural damage characteristics. Even with identical process parameters, the possibility that differences in thermal history due to strut thickness variations affected the microstructure cannot be ruled out.

### 4.4. Energy Absorption Characteristics

The evaluation of energy absorption performance mainly includes two aspects: energy absorption capacity and efficiency of absorbing energy. Energy absorption capacity refers to the energy absorbed per unit volume of a structure [52,53,54], also known as volumetric energy absorption:

(4) $$\omega =\int_{0}^{\varepsilon_{a}}\sigma (\varepsilon )d\varepsilon$$

**Figure 13:**
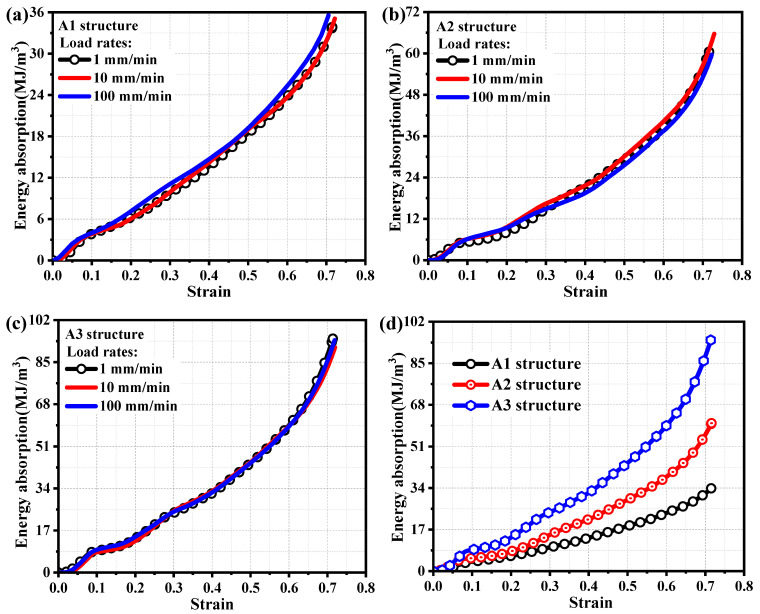
(**a**) Energy absorption capacity of A1 structure at different loading rates; (**b**) energy absorption capacity of A2 structure at different loading rates; (**c**) energy absorption capacity of A3 structure at different loading rates; (**d**) energy absorption characteristics of different structural volume fractions.

In the formula, *ε_a_* represents the structural strain, and *σ*(*ε*) represents the corresponding stress when the structural strain is *ε_a_*. Figure 13 shows the energy absorption capacity of porous metal structures at different loading rates. From Figure 13a, it can be seen that the energy absorption capacity pattern of the A1 structure is basically similar at different loading rates, and different loading rates have little effect on its energy absorption capacity. From the analysis of Figure 13b,c, it can also be seen that the energy absorption capacity of A2 and A3 structures is not affected by the loading rates. Although the energy absorption capacity of A1, A2, and A3 structures is not affected by loading rates, they are more significantly affected by the volume fraction of the structure. The A1 structure with the minimum structural volume fraction (9.43%) has the smallest energy absorption capacity. The A2 structure (15.83%), which has a higher volume fraction than the A1 structure, has significantly better energy absorption capacity than the A1 structure. Similarly, the energy absorption capacity of the A3 structure with the maximum structural volume fraction (22.15%) is significantly enhanced compared to the A2 structure, as shown in Figure 13d.

Efficiency of absorbing energy refers to the ratio of the total energy absorbed by a porous metal structure to the stress value [55,56]:

(5) $$\eta (\varepsilon_{a})=\frac{\int_{0}^{\varepsilon_{a}}\sigma (\varepsilon )d\varepsilon }{\sigma (\varepsilon ){|}_{\varepsilon =\varepsilon_{a}}}$$

**Figure 14:**
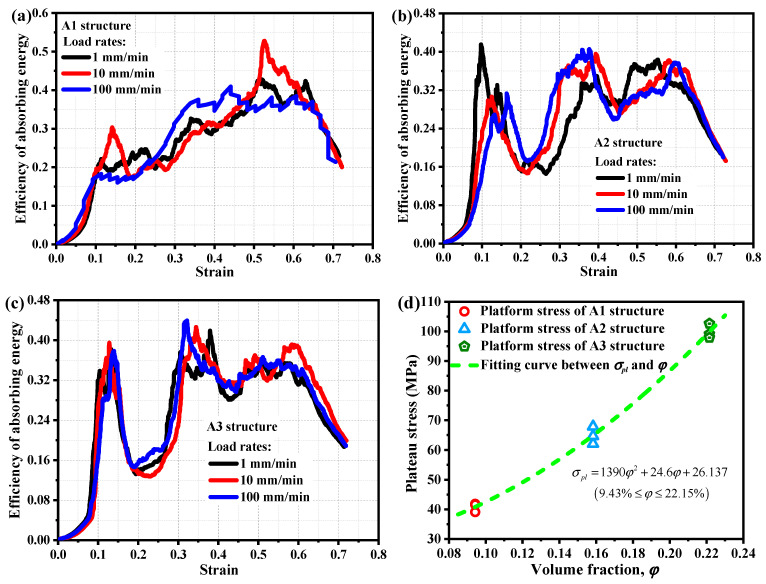
(**a**) Efficiency of absorbing energy of A1 structure at different loading rates; (**b**) efficiency of absorbing energy of A2 structure at different loading rates; (**c**) efficiency of absorbing energy of A3 structure at different loading rates; (**d**) distribution of platform stress for A1, A2, and A3 structures.

The higher the efficiency of absorbing energy, the better the energy absorption effect. Figure 14a–c show the energy absorption efficiency states of A1 structure, A2 structure, and A3 structure at different loading rates, respectively. As shown in the figure, the loading rate has little effect on the energy absorption efficiency characteristics of porous metal structures, indicating that there is no significant loading rate effect on their energy absorption efficiency.

Compaction strain, also known as densification strain, *ε_d_*, is the physical quantity that represents the strain corresponding to the maximum efficiency of absorbing energy of the end stress platform, that is,

(6) $$\frac{d\eta (\varepsilon )}{d\varepsilon }{|}_{\varepsilon =\varepsilon_{d}}=0$$

Stress analysis of porous metal structure platforms is an important research topic in the field of materials science, which helps to understand the mechanical behavior of materials under complex loading conditions [57,58,59,60,61,62]. The platform stress of materials and structures can be defined as

(7) $$\sigma_{pl}=\frac{\int_{\varepsilon_{s}}^{\varepsilon_{d}}\sigma (\varepsilon )d\varepsilon }{\varepsilon_{d}-\varepsilon_{s}}$$

In the formula, *σ_pl_* is the platform stress of the material or structure, and *ε_s_* is the yield strain. Figure 14d shows the distribution of platform stress for A1, A2, and A3 structures. Based on the volume fraction content of porous metal structures and the analysis of their platform stress distribution, it is found that the platform stress of porous metal structures is not linearly related to their volume fraction. The following correlation form is proposed to be established:

(8) $$\sigma_{pl}=\lambda_{1}\varphi^{2}+\lambda_{2}\varphi +\lambda_{3},9.43\%\leq \varphi \leq 22.15\%$$

In the equation, *φ* is the volume fraction of the structure, and *λ*_1_, *λ*_2_, and *λ*_3_ are polynomial constants. The values of constants *λ*_1_, *λ*_2_, and *λ*_3_ were determined based on the distribution of platform stress for A1, A2, and A3 structures with different volume fractions, which were 1390, 24.6, and 26.137, respectively. As shown in Figure 14d, the established intrinsic correlation model (Equation (8)) between the stress of the porous metal structure platform and the volume fraction of the structure can well describe the interaction and correlation mode between the stress of the porous metal structure platform and the volume fraction of the structure.

**Figure 15:**
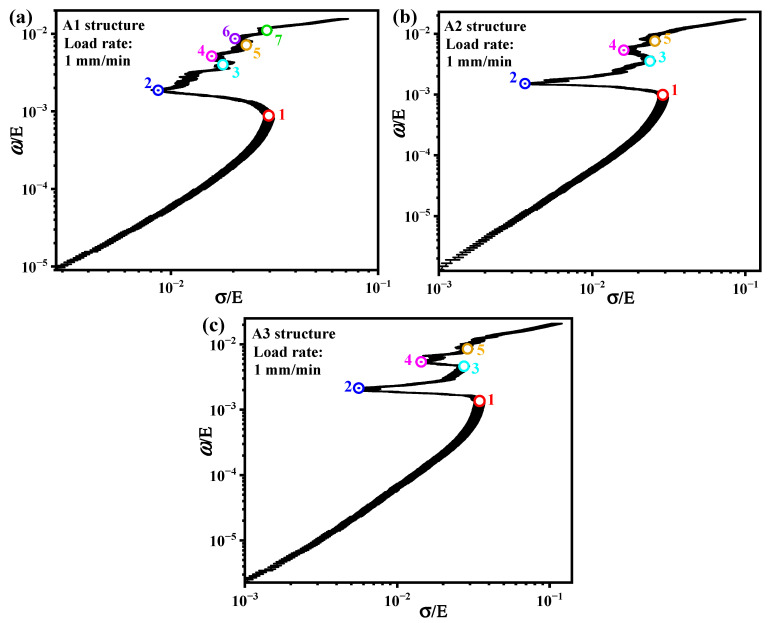
Normalization results of energy absorption capacity per unit volume and stress state of (**a**) A1 structure, (**b**) A2 structure, and (**c**) A3 structure at a loading rate of 1 mm/min.

To provide a basis for engineers to optimize the design of porous metal structures, it is necessary to normalize the energy absorption capacity per unit volume and stress state of porous structures separately. The elastic moduli (E) of A1, A2, and A3 structures were used to normalize the energy absorption capacity per unit volume and stress state of the structures, respectively. The normalization results are shown in Figure 15. The numbers 1, 3, 5, and 7 in Figure 15a represent the four stress peak points of the A1 structure before densification, and also indicate that at the position of number 1, the A1 structure reaches the first bearing limit and then begins to damage and break, while numbers 3, 5, and 7 begin to show new damage. These four positions are the stress-sensitive points of the A1 structure. The positions of numbers 2, 4, and 6 indicate temporary damage evolution in the structure, with a slow increase in load-bearing capacity until new damage occurs. The numbers 1, 3, and 5 in Figure 15b,c are the stress-sensitive points of the A2 and A3 structures, and temporary damage evolution occurs in the structures at numbers 4 and 6. If a large number of porous structures are combined with multiple testing and analysis modes to establish an intuitive graph of the analysis results, it can help structural design engineers choose the most suitable materials or structures for different service requirements.

## 5. Conclusions

In this paper, a combination of experimental research, numerical simulation techniques, and theoretical analysis was used to systematically elucidate the mechanical behavior and response mechanism of porous metal structures in titanium alloys. The following main conclusions were obtained:

(1) Based on a quasi-static compression experimental setup, a high-precision digital image correlation (DIC) technique combined with in situ mechanical testing was used to successfully obtain the stress–strain curves of titanium alloy porous metal structures at different loading rates. It was also found that the mechanical behavior of the structure was insensitive to loading rates but more sensitive to its structural volume fraction. From a 9.43% volume fraction of the A1 structure to a 22.15% volume fraction of the A3 structure, its bearing strength increased sharply from 64.5 ± 1.9 MPa to 157.9 ± 4.7 MPa.
(2) High-precision numerical simulation technology has been developed, which clarifies that the overall stress distribution of the titanium alloy porous metal structure is relatively uniform without obvious stress concentration, indicating that the structural design has good mechanical adaptability and engineering feasibility. The damage modes and damage evolution laws of porous metal structures in titanium alloys have been comprehensively elucidated. The numerical simulation results are basically consistent with the experimental results, indicating that this numerical simulation study is accurate and effective, including finite element models, material models, mechanical models, etc. It can provide an important theoretical basis and a reliable database for the study of the damage mechanism of the structure.
(3) Based on the analysis of microstructure fracture morphology, small and deep ductile dimples were found in the shear fracture surface, and some even exhibited stacking and overlapping phenomena, indicating that the material underwent plastic deformation before fracture. In addition to the toughness mechanical properties, a large area of brittle-like characteristics fracture mechanics behavior was found on the fracture surface, indicating that damage fracture occurred in this region at extremely low strains, resulting in brittle fracture. The quantitative characterization of microstructure damage and fracture morphology, as well as the identification of failure modes, indicates that the microstructure damage of the porous metal exhibits a ductile–brittle synergistic damage characteristic.
(4) By analyzing energy dissipation and constructing evaluation indicators for energy absorption efficiency, the energy absorption characteristics of porous metal structures were elucidated, and it was clarified that there was no significant loading rate effect. The established intrinsic correlation model between stress and structural volume fraction in porous metal structure platforms can effectively describe the interaction behavior and correlation mode between stress and structural volume fraction in porous metal structure platforms. Normalize the energy absorption capacity per unit volume and stress state of porous metal structures separately, and provide their structural stress-sensitive points, which provide an important basis for engineers to optimize the design of porous metal structures.

## Figures and Tables

**Table 1:** Composition percentage of Ti6Al4V powder in this paper.

Alloying Element	Ti	Al	V	Fe	O	C
Wt%	rest	5.7~6.3	3.8~4.1	≤0.3	≤0.2	≤0.1

**Table 2:** Parameters of Johnson–Cook constitutive model.

*A*/MPa	*B*/MPa	*C*	*n*	*m*	${\dot{\varepsilon }}_{0}$/s^−1^	*T_r_*/K	*T_m_*/K
1060	1090	0.0117	0.884	1.1	4 × 10^−4^	293	1878

**Table 3:** Parameters of Johnson–Cook fracture criterion.

*D*_1_	*D*_2_	*D*_3_	*D*_4_	*D*_5_	ρ/kg·m^−3^	E/GPa	ν
−0.105	0.27	0.48	0.014	3.87	4430	115	0.33

## Data Availability

The original contributions presented in this study are included in the article. Further inquiries can be directed to the corresponding author.
